# Drinking Water Quality Status and Contamination in Pakistan

**DOI:** 10.1155/2017/7908183

**Published:** 2017-08-14

**Authors:** M. K. Daud, Muhammad Nafees, Shafaqat Ali, Muhammad Rizwan, Raees Ahmad Bajwa, Muhammad Bilal Shakoor, Muhammad Umair Arshad, Shahzad Ali Shahid Chatha, Farah Deeba, Waheed Murad, Ijaz Malook, Shui Jin Zhu

**Affiliations:** ^1^Department of Agronomy, College of Agriculture and Biotechnology, Zhejiang University, Zijingang Campus, Hangzhou 310058, China; ^2^Department of Biotechnology and Genetic Engineering, Kohat University of Science and Technology, Kohat 26000, Pakistan; ^3^Institute of Soil & Environmental Sciences, University of Agriculture, Faisalabad, Faisalabad 38040, Pakistan; ^4^Department of Environmental Sciences and Engineering, Government College University, Allama Iqbal Road, Faisalabad 38000, Pakistan; ^5^Institute of Home and Food Sciences, Government College University, Faisalabad, Pakistan; ^6^Department of Applied Chemistry, Government College University, Faisalabad, Pakistan; ^7^Department of Botany, Kohat University of Science and Technology, Kohat 26000, Pakistan

## Abstract

Due to alarming increase in population and rapid industrialization, drinking water quality is being deteriorated day by day in Pakistan. This review sums up the outcomes of various research studies conducted for drinking water quality status of different areas of Pakistan by taking into account the physicochemical properties of drinking water as well as the presence of various pathogenic microorganisms. About 20% of the whole population of Pakistan has access to safe drinking water. The remaining 80% of population is forced to use unsafe drinking water due to the scarcity of safe and healthy drinking water sources. The primary source of contamination is sewerage (fecal) which is extensively discharged into drinking water system supplies. Secondary source of pollution is the disposal of toxic chemicals from industrial effluents, pesticides, and fertilizers from agriculture sources into the water bodies. Anthropogenic activities cause waterborne diseases that constitute about 80% of all diseases and are responsible for 33% of deaths. This review highlights the drinking water quality, contamination sources, sanitation situation, and effects of unsafe drinking water on humans. There is immediate need to take protective measures and treatment technologies to overcome unhygienic condition of drinking water supplies in different areas of Pakistan.

## 1. Introduction

Pakistanis are situated in southern Asia, bordering with India in the east, Afghanistan in the west, and China in the north. In the east of Pakistan, there exist mountains of Himalaya and Karakorum. In the north, Hindukush ranges exist, and hill regions (up to 4700 m) in the northwest and in the upland Baluchistan plateau exist. The climatic conditions are mostly arid to semiarid with varying levels of average rainfalls in different areas of Pakistan [1]. Indus is the major river of Pakistan, flowing from Karakorum ranges to south and finally falls in the Arabian Sea. Agriculture farming has a major role in Pakistan's economy. 27% of the total land is under farming and the main crops are wheat, maize, rice, cotton and sugarcane. To fulfill the requirements of increasing population, pesticides, and fertilizers are applied to increase the crops outcome. Most of industries such as textile, pesticide, and fertilizer industries are present in major cities.

Pakistan has been blessed by nature with enough surface and groundwater resources. Industrialization, urbanization, and rapid population growth have placed huge stress on water resources [2]. Water has a vital role in our life processes including growth and development. It plays significant role in our every field of life [3]. Due to technological developments, drinking water may contain various impurities, which are of physical, biological, and chemical nature. The most dangerous impurity is of biological nature, which causes human health problems or cause death [4]. Various impurities in the form of nutrient and microorganisms are transported from one place to another [5]. Water pollution occurs when microorganisms and toxic chemicals from domestic waste and industries either come in contact with water bodies or run off or leach into ground water or freshwater resources [6]. The contamination of animal and human fecal indicates presence of coli form bacteria [7]. The growth and dispersion of bacteria is at peak in rainy season due to drainage in water bodies, that is, rivers, lakes, and streams. Poor treatment facilities cause spread of waterborne diseases. In Pakistan, drinking water sanitation system and drainage lines run in parallel, which causes leakages and intermixing result in deterioration of water quality [8].

In most of the cities of Pakistan, the elementary source of provision is ground water supply, which contains various pathogens including many viral, bacterial, and protozoan agents causing 2.5 million deaths from endemic diarrheal disease each year [9].

Water pollution is a physical process that occurs in various water resources such as lakes, ground water, and rivers due to anthropogenic activities [10]. The utilization of poor quality water causes waterborne diseases and their spread. In Pakistan, about 50% of diseases and 40% of deaths occur due to poor drinking water quality reported in community health studies [11]. Above 80% of the people of province of Khyber Pakhtunkhwa (KP) are utilizing clean drinking water that comes from surface and ground sources. In KP, surface water resources are clean and fit for drinking but, in the south, color of ground water is blackish. Clean water is also found in deepness. But, in the center of KP ground water availability is excessive and pumped out with the help of tube wells for routine use. The water resources in the north of KP are mainly surface water resources and springs. The drinking water quality and quantity are very low because of poor treatment of deteriorated water and old sanitation system in urban areas [12].

When water comes from surface water resources, it is necessary to make it safe for drinking purposes. Chlorination is the popular method for disinfection of drinking water at treatment plant and in sanitation system [35]. It is the universal method to make drinking water safe and to reduce epidemic diseases [36–39].

Drinking water should be free from color, turbidity, odor, and microbes. It should be esthetically pleasant. Due to increasing population of Faisalabad, contaminated water is the most alarming problem. In 1999, Faisalabad required about 64.7 million gallons of drinking water supply daily to fulfill needs. But unfortunately 3 million gallons of this water came from domestic pumps that come out from subsoil water and tube well [40]. According to Pakistan National Conservation Strategy [41], less rain, drought, and nondevelopment of other water resources reduce water availability and increase water scarcity.

The current water supply is about 79% in Pakistan [42]. Improper and poor water supply for drinking purpose has a great health risk to the public. The release of toxic chemicals from urban communities and industries without any treatment into water bodies deteriorates water quality and also causes adverse effects to human beings. In Pakistan, water and sanitation agency has been focusing on water quantity due to increasing requirements rather than water quality. All this is due to the lack of awareness, treatment technology, equipment, trained personnel, and quality monitoring [43].

Human health is adversely affected by various agents like pathogens, bacteria, various minerals, and organic substances that are present in unsafe drinking water. A significant proportion of population in developing countries is suffering from health-related issues due to unsafe drinking water and microbial contamination [44]. In the developing countries, about five million children deaths occurred due to the contaminated drinking water supply [45]. This situation is intensifying day by day due to the fast population growth which ultimately results in poor management of water quality [46]. It is estimated that, in Pakistan, 30% of all diseases and 40% of all deaths are due to poor water quality [47]. Diarrhea, a waterborne disease, is reported as the leading cause of death in infants and children in Pakistan while every fifth citizen suffers from illness and disease caused by the polluted water [48].

In Pakistan, approaches to safe drinking water have reached acceptable limits. Reference [49] analyzed that about 25% population has approach to safe drinking water. The poor water supply was caused by the lack of water availability. Water pollution is mainly caused by heavy utilization of water for domestic, agricultural, and industrial purposes.

In Pakistan, the main reasons of waterborne diseases in drinking water are the addition of municipal sewage and industrial wastewater at different points of the water distribution network as well as lack of water disinfection and water quality monitoring at treatment plants. Pakistan National Conservation Strategy [41] reported that water-related diseases represent 40% of the communicable diseases. In Pakistan, waterborne diseases are typhoid, giardiasis, intestinal worms, diarrhea, cryptosporidium infections, and gastroenteritis. Infant deaths caused by water-related diarrhea are 60% in Pakistan according to International Union on Conservation of Nature (IUCN) report, which is the highest ratio in Asia.

In Pakistan, water quality in most of the cities is decreasing quickly. The major cause of decreasing water quality is the ground water supply. According to [50], the number of diarrheal cases that are registered in Pakistan each year is about one hundred million. According to Tahir et al. [51], above eighty thousand cases related to waterborne diseases were noted in healthcare units only in Rawalpindi. 20%–40% of hospitals of Pakistan are filled with people that are suffering from waterborne illness, according to United Nation International Children Emergency Fund (UNICEF). Diseases such as cholera, typhoid, dysentery, hepatitis, giardiasis, and cryptosporidiosis and guinea worm infections represent about 80% (including diseases due to sanitation problem) of all diseases and are responsible for 33% of deaths [51].

## 2. Water Availability

**Figure 1:**
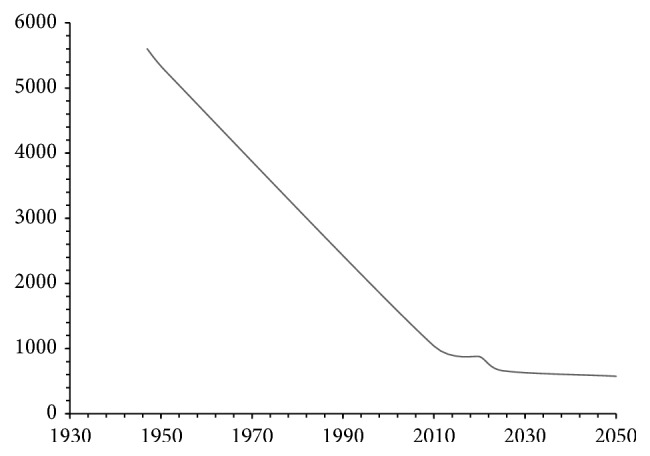
Per capita water availability decreasing tremendously in Pakistan.

Nature has gifted Pakistan with enough ground and surface water resources. Unfortunately anthropogenic activities such as industrialization, increase in population, and improper utilization decrease the quantity and damage the quality. According to Jamshed Iqbal Cheema (Chairman: Pakistan Agriculture Scientists Association), the per capita water availability in Pakistan at the time of independence was 5,600 cubic meters [52], which has been decreased by over 406 percent from 5,260 cubic meters in 1951 to 1,038 cubic meters in 2010. If the status quo continues, then, by 2020, the water availability in Pakistan will further plummet to 877 cubic meters per annum and will further decrease to 660 by year 2025 and will further go down to an alarming level of 575 cubic feet in 2050 [53] (Figure 1).

**Figure 2:**
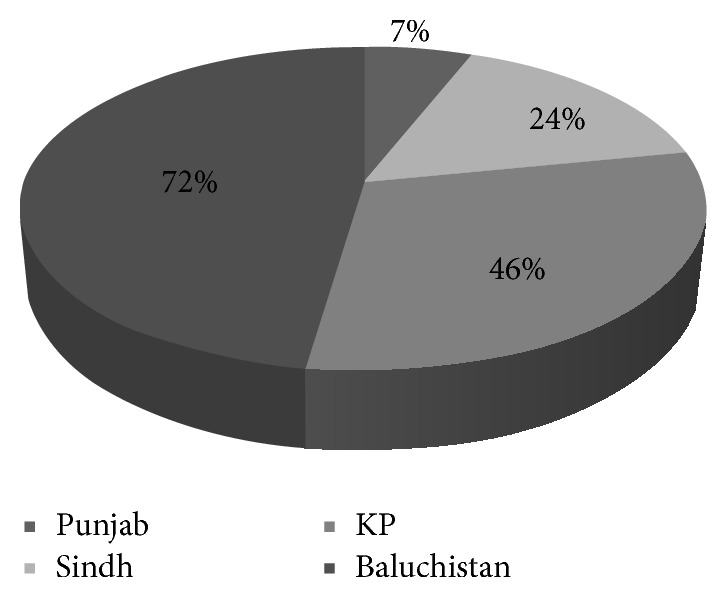
Dependence of water supply in the provinces of Pakistan.

In Punjab, 7% of all the rural population depends on dug wells and rivers for water supply. It seems that Punjab has best water supply system among all the provinces. This ratio is 24% in Sindh and people are utilizing water from unprotected sources. The rural communities of Khyber Pakhtunkhwa (KP) and Baluchistan using water from surface and dug well are about 46% and 72%, respectively, [1], as shown in Figure 2.

## 3. Water Quality

Water for drinking purposes mainly comes out from the surface and underground aquifers near the rivers or canals. The surface water quality is dropping rapidly due to the addition of raw municipal and industrial effluents and agriculture runoff into water resources [54]. When flow of river water is at its peak, it contains high solid suspension. Most of the rivers are extended and diluted and do not endure aquatic life. It is clear that these water bodies are fecally contaminated and need proper processing to free them from contaminants for human use. In Pakistan, four major cities have been using surface water; these are Islamabad, Karachi, Rawalpindi, and Hyderabad.

About 70% of water for drinking purposes comes from aquifers [55]. The decrease in ground water quality is due to the overpumping of saline water and its addition to fresh water. The ground water quality in Pakistan is found saline far away from the main rivers and fresh water near to the main rivers. The quality of drinking water is determined by the quality of water source, the level and treatment efficiency, and condition of water supply lines. In Pakistan, in most areas where the fresh water source is not available and ground water is saline, people have no choice but to use this type of water for drinking. The contamination of water due to microbes is the most blistering issue. The drinking water distribution in urban areas does not meet the WHO standards [56]. The main reason of microbial contamination is due to the intermixing of sewer lines with drinking water supply lines. In most of the rural areas of Pakistan, surface water is used for drinking after slow sand filtration and chlorination is not done at filtration stations. In most rural areas, no pretreatment facilities are available for filtration of water. All this inadequacy is due to microbial contamination and poor water quality. Hand pumps and wells are not safe from surface runoff and flooding [57].

Water pollution is the deterioration of water quality due to the addition of wastes coming from industries, domestic and agriculture. Utilization of such water for beneficial use causes contrary effects on environment and public health. Industrialization and emergence of urban units placed immense stress on water resources and discharge of wastewater into natural water resources that decreases ground and surface water quality [58].

The most serious pollutants in terms of human health worldwide are pathogenic organisms. Altogether, at least 25 million deaths each year occur due to these water-related diseases, including nearly two-thirds of the deaths of children under five years of age. The main and major source of biological agents is unprocessed and unconventional treatment of human waste [59]. The highest infant mortality rate (12.6%) and fertility rate (7%) reflect the poor health status of Pakistan. The bare hospital information indicates that most of the treated diseases are due to fecal contamination. About 25% of patients treated at hospitals, private clinics, or healthcare centers are suffering from diarrhea including children and adults [60, 61].

## 4. Water Quality Parameters

The physical, biological, and chemical properties of drinking water have great importance because a minor fluctuation in these parameters affects the human health. The pH is crucial factor that greatly affects water quality and quantity of pollution in water bodies [62]. However, pH of drinking water has no direct effect on human. Indirectly it changes meat solubility and provides suitable environment for pathogens. High pH causes acidic taste of drinking water [63]. The defined standards of drinking water quality [64] are shown in Table 1.

## 5. Sources of Contamination

### 5.1. Microbiological Contaminants

In Pakistan, microbial pollution has been discovered as one of the serious problems in rural as well as urban areas. This is due to the leakage of pipe, pollution from sewage lines intrusion into drinking water supplies, and so forth.

### 5.2. Chemical Contaminants

Chemical contaminants come from industries, soil sediments, and runoff from agriculture, that is, pesticides and fertilizers, and enter into water resources. In Pakistan, the application of fertilizer and pesticides is, respectively, about 5.6 million tons and 70 thousand tons according to Gross Operating Profit (GOP) figures. These chemicals, commonly insecticides, leach into ground water resources by mixing with irrigated and rain water. During 1988–2000, about 107 samples were collected from ground water and 31 samples indicated pesticide contamination that was clearly beyond the Food and Agriculture Organization (FAO) and WHO permissible limits. In Pakistan, another important trouble with ground water is highest concentration of salts, which is mainly due to irrigation, soil salts dissolution, sea water encroachment, and chemical industries. Salinity impacts the major areas of Baluchistan, KP, and Punjab. Effluent from industries and domestics contains high concentration of arsenic that is becoming a severe problem. In major cities of Sindh and Punjab, about sixteen percent of people are exposed to more than 50 ppm of arsenic. Higher concentration of fluoride above permissible limits causes a trouble in major areas of Baluchistan, Punjab, and Sindh. The dental fluorosis diseases are commonly found in Sindh, Punjab, and KP.

## 6. Floods Cause Major Damage to Drainage System

In Pakistan, floods have been creating great environmental problems. They damage drains and ultimately cause spillage of sewage water into water bodies. Severe flooding destroys buildings and standing crops. All these may cause release of toxic chemicals and oil into river, streams, and lakes, and so forth and may lead to death of aquatic life. A lot of chemical contaminants mix with flood water on its way. The current severe flood (2010) and heavy rains damaged 80% of Nowshera, devastating 40% of infrastructure. The total destroyed and damaged houses were in the range of 10,000 and 40,000, respectively [65].

## 7. Water Quality Status in Provinces of Pakistan

### 7.1. Water Quality Status in Twin Cities

To evaluate the drinking water quality of Islamabad, drinking water samples had been collected from schools and colleges. Analysis showed that 20 samples out of 30 were contaminated with fecal microbes and not fit for drinking purposes [66]. Microbial contamination is the most common and widespread risk associated with drinking water. About 130 samples were collected from nine areas to analyze microbial contamination in drinking water of Rawalpindi and Islamabad. 56.1% of water samples were found to have microbial contamination. Microbial contamination for fecal coliforms,* E. coli*, and total coliforms was 23.8%, 20%, and 12.3%, respectively. The WASA supply lines were highly contaminated followed by capital development authority lines and boring water and less contamination was found in tanker water [16], while thirty-two samples were collected from different water filtration plants throughout Islamabad city and it was found that more than half of the samples were contaminated with total coliform, fecal coliform, and* E. coli* [18].

Geographic Information System and Water Quality Index study of bore wells and open wells of Rawalpindi and Islamabad revealed that more than half of samples were poor in quality for drinking due to overexploitation of groundwater resource, agricultural impact, and direct release of contaminants [15].

Drinking water contamination with* E. coli* and fecal coliforms is clear indication of human and animal waste intervention [67]. In Rawalpindi, water distribution channels and treatment plants were also having fecal coliform contamination [67, 68]. The Rawal Lake and its distributions channels are the main source of drinking water for Rawalpindi, which were also found highly contaminated with bacteria [69]. Water quality of Islamabad was analyzed by [13]. Results obtained showed that about 77% of the total 271 samples collected were biologically contaminated and unfit for human use. On the other hand 10.3% of the total samples were found to be physically and also biologically contaminated, among which 196 samples from capital development authority (CDA) were collected for drinking water analysis. The result showed that 5.1% of the total samples collected were found to be bacteriologically contaminated and 3.6% were found to be both physicochemically and bacteriologically contaminated. In Islamabad and Rawalpindi, the water quality was not found better than the whole country. The water quality of natural streams situated in the capital city is also deteriorated. Water reservoirs were highly contaminated with total coliform and fecal coliforms bacteria, so proper water treatment for drinking and domestic use is required [70]. The heterotrophic bacterial assessment of drinking water quality of tube wells, water supplies, and filtration plants in various sectors of Islamabad revealed that 21% of 55 samples were contaminated with total coliform, fecal coliform, and* E. coli* [17].

Physicochemical parameters on water quality of Islamabad indicated that alkalinity, hardness, and total dissolved solids in all samples were within safe limits as recommended by Pakistan Standard and Quality Control Authority (PSQCA). But coliform and* E. coli* were detected in all water samples, so water was found unfit for drinking purposes as WHO recommended [14]. Higher amount of calcium, lime stone, and magnesium carbonate in drinking water caused significant level of hardness in I-9 and G-10 sectors, in Islamabad [71].* E. coli* was detected in drinking water samples collected from Risalpur, Pabbi, and Tarnab [72] as shown in Table 2.

### 7.2. Water Quality Status in Punjab

Drinking water quality and chlorination effect of two villages in south Punjab were analyzed [73]. Results of this study highlighted that all 53 samples collected from two villages had significant numbers of* E. coli* bacteria before and after chlorination process. According to WHO and PEPA, drinking water should contain 0/100 mL of* E. coli* or coliform.

Faisalabad is known as polluted industrial city due to the inadequate treatment facilities. The impact on water resources near Samundri drain in Faisalabad showed that the ground water quality was the worst as 90% of samples were above the WHO limits with respect to TDS, Na, K, Cl, and SO_4_ [21]. The people's perception of rural areas in a tehsil Samundri, district Faisalabad, was that the water quality of different sources, that is, hand and electric pumps, was poor [26].

The physicochemical analysis of drinking water was carried out to evaluate drinking water quality of Faisalabad city. The turbidity, hardness, pH, and TDS were found within safe limits of WHO guidelines. The microbial analysis showed that all samples were contaminated with total coliforms and* E. coli* [22]. The impact of municipal and industrial wastewater on water resources in Faisalabad showed that the physicochemical properties of ground water were beyond the critical values of WHO. However, bottle and supply lines were within critical range [20].

Chemical and biological analyses of drinking water samples collected from three different sites in Faisalabad showed that pH was found within the range according to WHO standards and electrical conductivity was found above the permissible limits. Higher electrical conductivity (EC) is due to the dissolution of subsoil minerals and leaching into ground water. Bacteria were also found in water samples which showed fecal contamination. All these analyses indicate that water is not fit for drinking purposes [74]. The concentration of As and coliform bacteria was above the threshold level in samples collected from different sources in University of Punjab, Lahore [19].

Water quality monitoring was carried out to access chlorination of supply lines in Cantonment area, Rawalpindi. The temperature of all samples was above the critical values as recommended by WHO. Water temperature is an important factor for microbial growth [75]. The pH and total dissolved solids are within range of US-EPA and WHO limits and similar to the results of [68]. Conductivity and chlorine residuals were also found within limits of WHO. Total dissolved solids and conductivity have a direct relation: as concentration of mineral salts increases, conductivity increases [76]. Microbial analysis indicated the presence of fecal coliform in all samples collected from both sites [77].

Drinking water quality of urban areas of southern Lahore was evaluated before and after monsoon season. It was seen that the values of pH of all sources and house connections were well within the WHO desirable limit both before and after the monsoon season. The turbidity in water was less than the desirable limit of 0.5 NTU while it was more than 0.5 NTU before and after the monsoon at two sites. The hardness at all the sources (T/W) and house connections was less than the WHO guideline. The TDS values were in critical limits. The bacteriological contamination was also not detected in water samples before and after monsoon. Fecal contamination showed that water had come in contact with human feces [78].

Drinking water quality test carried out in twelve districts of Punjab showed that microbial and heavy metal (arsenic) were major contamination found in all districts. At least 45% of the samples of Kasur district were found to be contaminated with microbes. About 73%, 100%, 64%, 94%, 100%, and 88% of drinking water samples of Sheikhupura, Lahore, Gujranwala, Multan, Kasur, and Bahawalpur were highly contaminated with arsenic. Total dissolved solids (TDS) were found above the permissible limits in Sargodha, Sheikhupura, Kasur, Faisalabad, and Rawalpindi [2]. Physical parameters of the samples collected from three different sites in Sabzazar district, Lahore, were within permissible limits of WHO. There was no detection of fecal coliform bacteria in samples collected from tube well and supply lines but* E. coli* contamination was detected in samples collected from household tabs showing that water was unfit for drinking purpose [79].

The chemical analysis of groundwater samples collected from rural areas of Punjab indicated that water was unfit for drinking purpose. High values of EC, Cl^−^, NO_3_^−^, SO_4_^2−^, Fe, Mn, and Pb were observed in many samples above the permissible limits [80]. The poor drainage system and improper waste dumping in villages of Pakistan are the main source of drinking water contamination. The bacterial analysis of drinking water samples of tube well, hand pumps, and turbines from Gangapur (village), Faisalabad, had a clear image of cow dung and municipal waste water contamination causing stomach diseases, that is, diarrhea, especially in infants [81].

The physicochemical analysis of different samples collected from urban areas of Faisalabad showed that the pH value and hardness were within range as recommended by WHO. The values of alkalinity, TDS, sulphate (SO_4_), and chlorides were found above the permissible limits of WHO. Overall the ground water used for drinking purpose in urban areas of Faisalabad was intensively polluted with sewerage water [82] as shown in Table 3.

### 7.3. Water Quality Status in Khyber Pakhtunkhwa (KP)

Water samples were collected from tube wells and storage tanks to determine the drinking water quality in rural areas of Peshawar. Results indicated that just 13% of the samples were negative for bacterial contamination, 40% were found in the satisfactory level, and 47% of the samples were found to be highly contaminated with* E. coli* [83]. The physicochemical analysis of drinking water samples collected from thirty different sites across urban areas of Peshawar described that pH at seven sites was not within WHO limits while EC was within range. TDS, turbidity, carbonates, and bicarbonates were within recommended range of WHO but magnesium was higher than critical level [84]. In districts Bannu and Haripur, physicochemical and microbial analyses of various portable water samples indicated that the water quality was poor and below the quality parameters of WHO [85, 86].

The drinking water samples collected for bacteriological detection demonstrated that about ninety-two percent of water samples were detected as contaminated [87]. The underground water quality of Swabi was analyzed for drinking purpose. Physical and chemical parameters of tube wells such as temperature, pH, EC, TSS, and BOD were within range of WHO [88]. The analysis of heavy metal contamination in drinking water of urban as well as rural areas of Peshawar described that the drinking water was highly contaminated with Pb and Cd. However, the concentrations of As, Cu, Co, Hg, Ni, and Zn were significantly higher than WHO limits making water unfit for drinking purpose. Therefore, there is urgent need to take steps to improve treatment technologies [23].

The physical and chemical parameters of drinking water in Narangi and sounding areas of Swabi district demonstrated that the physical parameters were within permissible limits but regarding chemical parameters Pb and nitrite concentrations were found higher than WHO limits [89]. The water quality of Nomal valley, Gilgit-Baltistan, indicated that the pH, temperature, turbidity, hardness, odor, taste, and alkalinity were within recommended range of WHO. But the microbial examination showed that all water samples were highly polluted [26].

Water samples collected for physicochemical analysis from tehsil of Jamrud and Landikotal, Khyber agency, showed that all parameters were within range set by WHO. However, the concentration of Ca and Mg exceeds the limits of WHO. The heavy metal concentrations were also below the WHO permissible limits. But Cd concentration was higher than WHO permissible limits [24].

Chemical and microbial aspects of water samples collected from four cities, that is, Abbottabad, Mardan, Peshawar, and Manghora, were analyzed. More than 55% of all samples from these cities were highly contaminated with microbes. In KP, iron contamination was enlisted as second major contamination. In Peshawar and Mardan more than 38% and 67% of samples were contaminated with iron, respectively [2]. The samples of drinking water collected from various reservoirs (streams, tube wells, and water storage tanks) in Kohat (KP) showed that samples collected from tube wells in Shakarda, Ara Khail, and Lachi were found to be safe for drinking but storage tanks and wells were highly contaminated. The drinking water quality of Charsadda (KP) was also poor as in other regions and indicated that the concentration of sulphate, nitrate, and heavy metals was above the threshold level and they were contaminated with coliform bacteria [90].

The microbial and physical investigation of drinking water quality in new urban Peshawar indicated that the pH was within permissible limits but the value of EC in five tube wells, seven supply channels, and nine storage tank samples was found above critical values. Similarly, the TSS values of water samples collected from supply channels, storage points, and tube wells were 30%, 60% and 10% beyond the critical limits of WHO. Bacteriological analysis showed that about one-third of all samples were not detected to have bacteriological contamination, while others were contaminated [25].

The water quality from different villages of Nagar valley revealed that all the tested parameters, that is, temperature, pH, turbidity, electrical conductivity, total dissolved solids, total coliform bacteria, total fecal bacteria, calcium hardness, cyanuric acid, and total alkalinity, were meeting the prescribed standards of WHO and EPA [91] as shown in Table 4.

### 7.4. Water Quality Status in Baluchistan

Biological and chemical water quality of Baluchistan are not satisfactory as revealed by various studies. In four cities of Baluchistan, that is, Ziarat, Loralai, Quetta, and Khuzdar, the water quality was badly contaminated with microorganisms making water unfit for human use. Water samples of these cities showed that NO_3_ concentration was higher than the recommended limits of WHO. About 50% of water samples, collected from Ziarat, were found highly contaminated with NO_3_ [2]. The drinking water quality assessment of different colonies in Quetta city revealed that pH, TDS, and hardness value of all samples were within the WHO range but 50% of the samples were found to have high EC value and COD of all samples was above the critical limits of WHO [29]. The drinking water quality of Quetta was inadequate having bad taste, foul smell/odor, change in appearance, and pathogens being 57%, 44%, 39%, and 60%, respectively [28].

Temperature examination revealed a little fluctuation in results between 12.10 and 13.50°C. The highest value was determined in Thole channel water while the lowest was found in Nilt tank water. According to WHO and EPA, turbidity must not exceed 5 NTU and water having turbidity less than 1.00 NTU is excellent for domestic consumption. Turbidity of all samples was less than 5 NTU [91]. The surface and groundwater sources of drinking water throughout Baluchistan were highly contaminated with coliforms, heavy metals, and pesticides. Human activities like improper disposal of municipal and industrial effluents and indiscriminate applications of agrochemicals in agriculture are the main factors contributing to the deterioration of water quality [92]. The fluoride concentration in various drinking water samples collected from tap and wells water in Quetta indicated that all samples were within permissible limits of WHO except one sample of tap water [93].

The bacteriological and physicochemical study of Hingol River situated at Hingol National Park was carried out, where the majority of its inhabitants are leading nomadic life style [94, 95] and consume the water of the river as no alternative water resources are available. The physicochemical parameters of the samples collected were according to the NSDWQ standards. But the TDS value was greater than the permissible limits in postmonsoon. The BOD concentration was also relatively higher [27] as shown in Table 5.

### 7.5. Water Quality Status in Sindh

The drinking water quality of Khairpur, Sindh, showed high level coliform and fecal coliform contamination in drinking water at different points; therefore, it is not suitable for drinking purpose. It is evident from the results (high coliform and fecal coliform count at all 3 levels) that the quality of drinking water is further deteriorated in the distribution system which may be due to the leakage of pipes where sewage water enters into the municipal water. At the consumer level, the drinking water is getting more contaminated due to the unhygienic handling and uncovered storage tanks. Drinking water quality should have no coliform as well as fecal coliform present in 100 mL or 0 colony forming units (cfu) per 100 mL WHO [96].

The ground water of different villages in district Khairpur, Sindh, was analyzed physicochemically for drinking and irrigation purposes. The chemical and physical characteristics of all samples were above the WHO guidelines and water was not fit for drinking as well as for irrigation purposes [30]. Bacteriological and physicochemical examination of groundwater in the coastal areas of Sindh indicated that groundwater was unfit for drinking purpose. Phosphate and sulphate concentrations were within range. But, organic and fecal contamination was higher than turbidity and salinity [33]. The ground water quality of various districts in Sindh showed that the pH of water samples was within limits of WHO, while turbidity and most of the chemical parameters were above the critical limits [32].

Water shortage is a major issue in Karachi city, which is worse in slum areas having poor infrastructure and limited facilities. The physicochemical analysis of drinking water supply lines in Orangi Town, Karachi, showed that physicochemical characteristics were within WHO permissible limits except sulphates. The microbial investigation revealed that all samples were highly contaminated with total coliform, fecal coliform, and* E. coli.* The presence of microbial contamination indicated poor water supply and sewage infrastructure [31]. Microbial and physicochemical parameters of water supplied by WASA in Gulshan-e-Iqbal demonstrated that the pH, temperature, turbidity, conductivity, TDS, and As were satisfactory to the guidelines of WHO but only three samples were contaminated with microbes because of leakage water mains and cross-connections between drinking water supply lines and sewage [34] as shown in Table 6.

Qualitative analysis of water resources that are used for drinking purposes showed that the physical parameters of three sampling sites such as bore well, dug well, and hand pumps were not according to the recommendations of PEPA and WHO [97]. But, however, samples collected from tube well were according to the PEPA and WHO [97] recommendations. Turbidity was found in the samples collected from hand pumps, bore well, and dug well but tube well water samples were found to be turbidity-free. The EC and contents of TS, TDS, and TSS were above the WHO [97] recommendations in the samples collected from hand pumps, bore well, and dug well. All these parameters of samples collected from tube well water were within WHO limits [65].

Drinking water quality of the Sindh province is also poor as that of other provinces. About 67%–93% of samples collected from different locations in three main cities, that is, Sukkur, Hyderabad, and Karachi, showed that water is unsafe for drinking purposes due to microbial and chemical contamination [2]. Guidelines for drinking water WHO [98] and National Standards for Drinking Water for Pakistan NSDWQ [99] recommend that* E. coli* or thermotolerant coliform bacteria must not be detectable in all water directly intended for drinking. However, total and fecal coliform bacteria were detected in samples collected from drinking water supply of Badin city and the water samples were found to be unfit for drinking [100].

The pH value of Keenjhar lake, located in Theta, Sindh, was within limits but color was brown to dark brown which is not acceptable for drinking purposes. The EC values of the samples were found to be above the WHO permissible limits.

## 8. Human Health Impacts

Due to the poor sanitation system, treatment, and monitoring, drinking water quality deteriorates. The presence of toxic chemicals and bacteria in drinking water causes adverse effect on human health. Due to the fecal contamination, people have been suffering from waterborne diseases. In rural and urban areas of Pakistan, cases of waterborne diseases, typhoid, dysentery, cholera, and hepatitis are systematically reported. However, it is very difficult to properly quantify the danger due to several reasons. They include underreporting of diseases and poor record maintenance in healthcare centers and hospitals related to diseases caused by poor water quality [101].

Several studies have reported health-related problems due to poor drinking water quality. For example, the concentration of nitrate (NO_3_) was found above the permissible limits causing blue baby syndrome in bottle fed babies [44]. The average daily intake of potassium (K) by adults was noted to be less than 0.1% through water [102]. Significant quantity of K is very important, the same as other elements for proper functioning of body. Diseases such as hypertension, kidney diseases, heart problem, muscle weakness, bladder weakness, and asthma may be caused due to K level decreasing in blood and increase in level may cause cysts, reduced renal function, rapid heartbeat, and improper metabolism of proteins [103]. The major source of sodium (Na) is the deposition of minerals into the water. Decrease in Na level in body causes low blood pressure, fatigue, mental apathy, and depression and increase in level may cause brain stroke, kidney problem, nausea, headaches, hypertension, and stomach problem [104]. Cardiovascular disease may be caused by the basic cations deficiency such as calcium (Ca) and magnesium (Mg) [105]. The basic and important element for myoglobin and hemoglobin and for numerous other enzymes is iron (Fe). The higher level of Fe in body also causes many health problems such as weakening of cardiovascular tissue, central nervous system, kidney, and liver, blood problems, vomiting, and diarrhea [106].

In Peshawar, most of water samples were found to be contaminated with coliform bacteria. In Rawalpindi, the gastroenteritis was reported in 2000; the contaminated water was the source. In Karachi, it was also found that the drinking water samples were heavily contaminated with total and fecal coliform. In Khairpur, a city of 0.12-million population, water quality seems to be poor and therefore could be a potential source for waterborne diseases especially among children.

In Islamabad and Rawalpindi, 4000 cases of hepatitis were registered and were due to unfit drinking water and improper treatment [107]. Dental fluorosis was also found in many districts such as Raiwind, Pattoki, and Kasur [43]. Effluents coming from tanneries contaminate the ground water in Kasur and cause skin and abdominal problems [108].

Unsafe drinking water is a major cause of the disease, which otherwise may be prevented, in particular in young children in developing countries. Pathogens present in drinking water including many viral, bacterial, and protozoan agents caused 2.5 million deaths from endemic diarrheal disease each year [9]. Major health problems were reported as gastroenteritis (40%–50%), diarrhea (47%–59%), dysentery (28–35%), hepatitis A (32%–38%), hepatitis B (16%–19%), and hepatitis C (6-7%) by respondents [29]. In southern Sind, waterborne diseases such as diarrhea, vomiting, gastroenteritis, dysentery, and kidney problem are caused by polluted drinking water [109].

## 9. Management Strategies

Management strategies should cover protection of sources from contamination, drinking water distribution lines upgradation and their proper maintenance, and monitoring and awareness of the people [110].

### 9.1. Legislative Control

There is a poor framework for the legislation of drinking water supplies. Drinking water quality standards should be provisionally established for the treatment and maintenance of drinking water distribution system. Water and Sanitation Agency (WASA) should take action with the help of private institutions to protect water resources and control pollution from its source. A great attention is also required to stop the saline water intrusion into the fresh ground water resources.

### 9.2. Governance

Government should take action for the maintenance, proper functioning, and handling of already present drinking water treatment plants. There is a lack of proper sampling system of the drinking water treatment plants to ensure that water is safe and fit for drinking in urban areas of Pakistan. To stop the spread of waterborne diseases, there is need for proper functioning, inspection, and sampling analysis twice a year to ensure safe drinking water according to the quality standards.

Proper maintenance of water distribution system and chlorination should be done according to the law and regulations to kill pathogens. Government should provide the latest and reliable instruments and trained personals for the drinking water quality analysis.

In Pakistan, there are few industries that have their own water treatment plant to treat wastewater. Government should take strict action for their industrial effluent disposal according to the NEQS under the 1997 Act. If any industry is found to be violating the rules, it should be punished with heavy fine and imprisonment.

### 9.3. General

Public awareness campaigns should be started at school, college, university, and community level to address the significance of secure drinking water. NGOs might act in this facet. Rural communities should adopt safe control methods for protecting water storage in houses and simple disinfection technologies of drinking water.

A lot of studies show that boiled drinking water reduces risk of waterborne diseases [111, 112]. A study was conducted by [113] in three districts of Punjab's urban as well as rural areas, that is, Toba-Tek Singh, Multan, and Rawalpindi. All the samples were collected through multistage sampling technique. The outcomes indicated that 45.1% of population of these three districts were not using National Quality Standards to improve water quality and these people were suffering from diarrhea. The remaining population of these three districts use National Quality Standards and were not found ill. Social and economic conditions of the families also play a vital role in reduction of diarrheal disease. It is also seen that mother's education, household income, and living style are correlated to the quality of drinking water and also improve health status of the family.

## 10. Conclusion

This review documented the studies conducted in Pakistan on drinking water quality status and contamination, which accounted sewerage water (fecal) mixing with drinking water as dominant and primary contaminant due to the poor sanitation and sewerage system. Second source of contamination is chemical pollution from toxic substances from the industrial effluents, textile dyes, pesticides, nitrogenous fertilizers, arsenic, and other chemicals. There is a need to maintain and upgrade regular inspection of already present treatment plants. Nowadays, Government of Pakistan is going to install drinking water filter all over Pakistan. The results drew attention that sewerage contamination with drinking water must be considered as an important environmental and health issue.

## Figures and Tables

**Table 1:** National Standards for Drinking Water Quality.

Parameters	Standard values for Pakistan	WHO standards
Biological		

All water intended for drinking (*E. coli* or thermotolerant coliform bacteria)	Must not be detectable in any 100 mL sample	Must not be detectable in any 100 mL sample
Treated water entering the distribution system (*E. coli* or thermotolerant coliform and total coliform bacteria)	Must not be detectable in any 100 mL sample	Must not be detectable in any 100 mL sample
Treated water in the distribution system (*E. coli* or thermotolerant coliform and total coliform bacteria)	Must not be detectable in any 100 mL sample In case of large supplies, where sufficient samples are examined, it must not be present in 95% of the samples taken throughout any 12-month period	Must not be detectable in any 100 mL sample In case of large supplies, where sufficient samples are examined, it must not be present in 95% of the samples taken throughout any 12-month period

Physical		

Color	≤15 TCU	≤15 TCU
Taste	None	None
Odor	None	None
Turbidity	<5 NTU	<5 NTU
Total hardness as CaCO_3_	<500 mg/L	—
TDS	<1000	<1000
pH	6.5–8.5	6.5–8.5

Chemical		

Essential inorganic	mg/L	mg/L

Aluminum (Al) mg/L	≤0.2	0.2
Antimony (Sb)	≤0.005 (P)	0.02

Arsenic (As)	≤0.05 (P)	0.01
Barium (Ba)	0.7	0.7
Boron (B)	0.3	0.3
Cadmium (Cd)	0.01	0.003
Chloride (Cl)	<250	250
Chromium (Cr)	≤0.05	0.05
Copper (Cu)	2	2

Toxic inorganic	mg/L	mg/L

Cyanide (CN)	≤0.05	0.07
Fluoride (F)^*∗*^	≤1.5	1.5
Lead (Pb)	≤0.05	0.01
Manganese (Mn)	≤0.5	0.5
Mercury (Hg)	≤0.001	0.001
Nickel (Ni)	≤0.02	0.02
Nitrate (NO_3_)^*∗*^	≤50	50
Nitrite (NO_2_)^*∗*^	≤3 (P)	3
Selenium (Se)	0.01 (P)	0.01
Residual chlorine	0.2–0.5 at consumer end, 0.5–1.5 at source	—
Zinc (Zn)	5.0	3

Organic		

Phenolic compounds (phenols) mg/L		≤0.002
Polyaromatic hydrocarbons (PAH) g/L		0.01 (by GC/MS method)

*∗* indicates priority health related inorganic constituents which need regular monitoring.

**Table 2:** Physicochemical and biological parameters of drinking water quality of twin cities.

Islamabad and Rawalpindi	Total	Percentage of samples contaminated										References
		pH	EC	TDS	Turbidity	Hardness	Physicochemical values	Total coliform	Fecal coliform	*E. coli*	Bacteriological values	
Islamabad (Isd.)	271	—	—	—	—	—	10.3	—	—	—	77	[13]
CDA (Isd.)	196	—	—	—	—	—	3.6	—	—	—	5.1	[13]
F-10, G-6, G-10, H-9, I-9, Dhok Kala khan, commercial market, New Mirpur, Pindora, Dhoke Ratta	10^a^	—	—	4^a^	—	4^a^	—	Present	Present	Present	—	[14]
Islamabad & Rawalpindi	22	—	—	—	—	—	46	—	—	—	—	[15]
Islamabad & Rawalpindi	130	—	—	—	—	—	—	Present	Present	Present	—	[16]
Islamabad	55	—	—	—	—	—	14.5	Present	Present	Present	—	[17]
Islamabad	32	—	—	—	—	—	—	Present	Present	Present	—	[18]

^a^Number of sampling areas.

**Table 3:** Physicochemical and biological parameters of drinking water quality of Punjab.

Punjab sampling locations	Total	Percentage of samples contaminated										References
		pH	EC	TDS	Turbidity	Hardness	Physicochemical values	Total coliform	Fecal coliform	*E. coli*	Bacteriological values	
University of Punjab, Lahore	18	—	—	—	—	—	11	Present	—	—	11	[19]
Faisalabad												
Bottled water		—	—	—	—	—	10					
Ground water	54	—	—	—	—	—	50	—	—	—	—	[20]
WASA supply lines		—	—	—	—	—	20					
Faisalabad	54	—	—	—	—	—	90	—	—	—	—	[21]
Faisalabad	225	—	—	—	—	—	—	Present	—	Present	79	[22]
Samundri, Faisalabad	110	—	—	—	—	—	—	Present	Present	Present	—	[16]

**Table 4:** Physicochemical and biological parameters of drinking water quality of Khyber Pakhtunkhwa (KP).

Khyber Pakhtunkhwa sampling locations	Total	Percentage of samples contaminated										References
		pH	EC	TDS	Turbidity	Hardness	Physicochemical values	Total coliform	Fecal coliform	*E. coli*	Bacteriological values	
Peshawar	74	—	—	—	—	—	Pb, Cd contamination	—	—	—	—	[23]
Khyber agency	50	—	—	—	—	—	Cd	—	—	—	—	[24]
New urban Peshawar	30	—	100	—	90	—	—	Present	Present	Present	—	[25]
Gilgit-Baltistan	27	—	—	—	—	—	—	Present	—	—	—	[26]

**Table 5:** Physicochemical and biological parameters of drinking water quality of Baluchistan.

Baluchistan sampling locations	Total	Percentage of samples contaminated										References
		pH	EC	TDS	Turbidity	Hardness	Physicochemical values	Total coliform	Fecal coliform	*E. coli*	Bacteriological values	
Hingol river, Baluchistan	22	—	12.5	—	—	—	—	Present	Present	—	—	[27]
Quetta	200	—	—	—	—	—	39	—	—	—	60	[28]
Khudar, Loralai, Quetta, and Ziarat	66	—	—	6	7.5	—	57.5	Present	Present	Present	80.3	[2]
Quetta	16	—	12.5	—	—	—	—	—	—	—	—	[29]

**Table 6:** Physicochemical and biological parameters of drinking water quality of Sindh.

Sindh sampling locations	Total	Percentage of samples contaminated										References
		pH	EC	TDS	Turbidity	Hardness	Physicochemical values	Total coliform	Fecal coliform	*E. coli*	Bacteriological values	
Khairpur, Sindh	68	—	50	20.5	—	13.11	—	—	—	—	—	[30]
Orangi Town, Karachi	46	—	—	100	—	—	39	Present	Present	Present	80.43	[31]
Sindh	35	—	—	50	—	21.88	—	—	—	—	—	[32]
Sindh	46	—	—	—	100	—	—	Present	Present	—	—	[33]
Gulshan-e-Iqbal, Karachi	12	—	—	8.3	—	—	—	Present	Present	Present	41.66	[34]
